# Integration of the serum metabolome and gut microbiome underscores the importance of altered lipid metabolism and potential immune modulation in Parkinson’s Disease

**DOI:** 10.1016/j.dscb.2026.100310

**Published:** 2026-02-21

**Authors:** Keren Zhang, Kimberly C Paul, Jonathan P Jacobs, Yuyuan Lin, Dayoon Kwon, Roch Nianogo, Jeff M Bronstein, Adrienne M Keener, Yu Yu, Aline Duarte Folle, Dean P Jones, Beate Ritz

**Affiliations:** a Department of Epidemiology, UCLA Fielding School of Public Health, Los Angeles, CA, USA; b Department of Neurology, UCLA David Geffen School of Medicine, Los Angeles, CA, USA; c The Vatche and Tamar Manoukian Division of Digestive Diseases, Department of Medicine, David Geffen School of Medicine at UCLA, Los Angeles, CA, USA; d Division of Gastroenterology, Hepatology and Parenteral Nutrition, VA Greater Los Angeles Healthcare System, Los Angeles, CA, USA; e UCLA Center for Health Policy Research, Fielding School of Public Health, University of California Los Angeles, Los Angeles, CA, USA; f Department of Medicine, School of Medicine, Emory University, Atlanta, GA, USA; g Department of Environmental Health Sciences, UCLA Fielding School of Public Health, Los Angeles, CA, USA

**Keywords:** Parkinson’s disease, Gut microbiome, Metabolome, Multi-omics, Lipid metabolism, Immune modulation

## Abstract

**Introduction:**

Single-omics studies have deepened our understanding of disease-related molecular processes, though integrated approaches are needed to uncover cross-system interactions. We previously reported changes in the serum metabolome and gut microbiome in Parkinson’s disease (PD). To build on these findings, we conducted a multi-omics integration analysis to examine the interplay between the gut microbiome and human metabolism in PD.

**Methods:**

In a community-based study of 113 PD patients from rural California, microbiome profiles were obtained via 16S rRNA gene sequencing of fecal samples. Serum metabolomic profiles were generated using untargeted high-resolution LC-MS. Residual matrices of metabolomics data were extracted after adjusting for age, sex, racial minority status, and study wave. We identified PD-associated bacterial genera and summarized their abundance using principal component analysis. Using this summary score as the dependent variable, we performed partial least squares (PLS) regression to identify serum metabolites associated with the PD-related gut microbiome. Pathway enrichment analysis was then conducted on selected metabolite features from the PLS model.

**Results:**

We identified 266 metabolite features and annotated 29 metabolic compounds associated with PD-related microbes (*p* < 0.1). Enrichment analysis revealed perturbed pathways in lipid metabolism, including fatty acid activation and metabolism, linoleate metabolism, and glycerophospholipid metabolism, as well as carbohydrate metabolism such as hexose phosphorylation and starch/sucrose metabolism.

**Conclusion:**

Our multi-omics integration analysis revealed that PD-associated gut microbiota are involved in host lipid metabolism, immune-related pathways, and potentially vitamin B-mediated regulation of kynurenine pathway metabolism, providing insights into potential microbiome–metabolome interactions in PD pathophysiology.

## 1. Introduction

Parkinson’s disease (PD) is a progressive neurological disorder that impacts multiple systems within the human body. While PD pathology is characterized by loss of dopaminergic neurons in the substantia nigra and accumulation of misfolded α-synuclein predominantly in the brain [1], these misfolded protein aggregates are also found in other tissues, importantly in the human gut [2,3]. The gut-brain axis is a concept that describes the bidirectional interaction between the gut and brain via the central and peripheral nervous system, the immune response, and endocrine systems [4]. The gut-brain axis has become a focus of PD research [5]. The development of omics technology has opened new avenues of research that may help unveil more information about this bidirectional communication [6–9], however, to date, very few human studies have had data available from multiple omics layers across which to integrate this information. Here, we present pilot study results with the primary goal to further the understanding of PD pathology at the molecular level, and also demonstrate the feasibility of a multi-omics approach in elucidating potential pathways of relevance in PD. From an epidemiologic case-control study of PD in rural California, we previously identified gut bacterial genera associated with PD [10]. Here, we further explored the gut microbial profile in this population and newly interrogated the serum metabolome to understand how these microbes, as a community, may contribute to human metabolism in PD patients.

## 2. Methods

### 2.1. Study population

This study utilized data collected in the Parkinson’s, Environment, and Gene study (PEG), a population-based study in rural central California investigating the etiology of PD. Participants were recruited in two waves: PEG1 from 2001 to 2007 and PEG from 2012 to 2017. At initial study enrollment, in Fresno, Tulare, and Kern counties, recently diagnosed PD patients (within the past 3–5 years) and non-PD community controls were randomly selected and recruited using tax assessor address lists. Follow-up visits were conducted for patients approximately every two years after baseline visits, which expanded the original case-control study into a PD cohort. From November 2017 to June 2020, we conducted a pilot study by inviting participants from the two prior recruitment waves to contribute fecal and blood samples. Additionally, we invited a household member of the PD patient to participate in the study. Participants were not included if they were immunocompromised or had taken any antibiotics within the past three months. All PD diagnoses and clinical characteristics were examined and confirmed by a study movement disorder specialist at baseline and follow-up visits. Trained research staff conducted interviews with the participants to collect information on demographics, dietary patterns using the Diet History Questionnaire (DHQ, created by National Cancer Institute), and daily levodopa dosage (among PD patients only) [11]. In total, we enrolled 211 participants in the pilot study (119 PD patients and 92 non-PD controls) with microbiome data, among whom 115 PD patients provided all data and biosamples necessary for metabolomic profiling in the current study. We further removed two samples from the analyses due to the large time gap between blood and fecal sample collection (> 10 years), resulting in a final analytic sample size of 113. This study was approved by the UCLA Institutional Review Board (#IRB-20-1352). Informed written consent was obtained from all study participants at initial study enrollment.

### 2.2. Gut microbiome assessment

Participants collected fecal samples using a Para-Pak^®^ self-collection kit. Fecal samples were immediately preserved in a vial containing 96 % ethanol and mailed to UCLA within 14 days of collection and stored at −80 ° C until DNA extraction. We used the ZymoBIOMICS DNA kit to extract bacterial DNA from fecal samples with bead beating. The V4 region of 16S rRNA genes was amplified and underwent pair-end 250 × 2 sequencing on Illumina platforms (HiSeq 2500 or MiSeq). We subsequently processed the raw sequencing data using the DADA2 (v 1.21.0) pipeline and phyloseq (v 1.34.0) packages in R, and further quality-filtered sequencing reads. Sequencing reads were processed into amplicon sequence variants (ASVs), a classification method that corresponds to taxonomy at species-level resolution. To assign taxonomy to these ASVs, we performed closed-reference taxonomic assignment using the Silva database. Due to the inconsistent taxonomic annotation depth at the ASV level, ASVs were further collapsed to the genus level for downstream analyses.

### 2.3. High-resolution metabolomics (HRM)

Serum samples were collected from participants during study visits and stored at −80 ° C until analysis. High-resolution metabolomics profiling was completed at Emory University according to established methods [12]. Detailed processing and quality control steps of the metabolomic data were described previously [13]. Briefly, samples underwent thawing and vortexing, followed by protein precipitation using acetonitrile. After centrifugation, the resulting supernatant was transferred to an autosampler. Two quality-control samples, NIST 1950 (analyzed at the beginning and end) and Q-Std (analyzed throughout), were used for normalization and batch effect evaluation. Samples were analyzed in batches of 40 using dual-column, dual-polarity HILIC chromatography with positive ESI and C18 chromatography with negative ESI. Features were represented by *m/z*, retention time (rt), and ion abundance. We further filtered the features by only retaining those with a median CV <30 %, Pearson correlation > 0.7, and presence in over 50% of samples, resulting in 2046 (C18) and 2716 (HILIC) features. Using the ComBat method, data were log2-transformed, quantile--normalized, and batch-corrected after zero values were replaced with the lowest detected value [14].

Two methods were used for feature annotation. First, features were matched against an internal reference library of confirmed compounds, allowing up to 20 ppm deviation for *m/z* values and 45 s for retention time differences. Second, xMSannotator was employed to associate features with public databases such as ChemSpider and HMDB [15]. This tool assigned a confidence score ranging from 0 to 3, and only those with scores of 2 or higher were included in the final dataset [15].

### 2.4 Statistical analysis

**Fig. 1.**
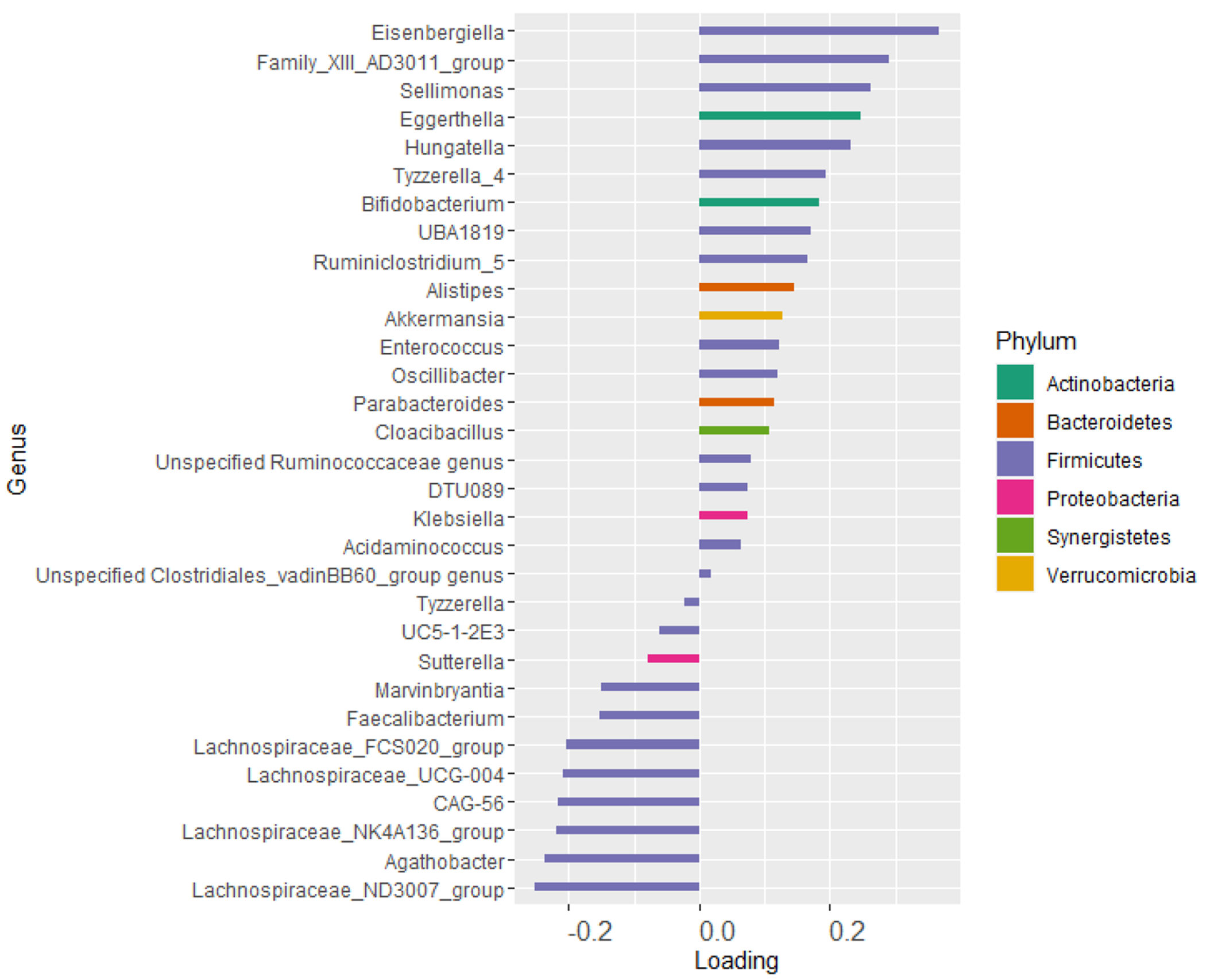
Loading of PD-related genera on PD bacterial score. Bar plot showing the loading of each PD-related genus on the PD bacterial score, defined as the first principal component from the principal component analysis of 31 genera.

In the previous report from the PEG study, we identified genera with differential abundance between PD patients and non-PD controls [10]. In the current analysis, with additional participants enrolled since the previous report, we replicated the analyses using the larger sample size (n = 211) and the MaAsLin2 package (v1.51.1), identifying 31 genera associated with PD (Table S1, Figure S1). Detailed methods of the analyses, including covariate adjustment and multiple testing correction, can be found in the previous report [10]. A summary “PD bacterial score” of these genera was generated using the first principal component (PC) from the principal component analysis (R package “PCAtools”, v 2.2.0) of the normalized microbiome data (R package “DESeq2”, v.1.30.0, for normalization) (Fig. 1, Table S2). We then restricted the analysis to PD patients who had undergone metabolomic profiling (*n*=113). Given the high dimensionality and strong correlations within the metabolomic data, we applied partial least squares (PLS) regression to identify metabolic features associated with the PD bacterial score. The metabolic features were first adjusted in regression analysis for potential confounders, including age, sex, racial minority, study wave, levodopa daily dosage, and PD duration (years since PD diagnosis). The residual matrix was used as the input matrix for the PLS regression of metabolites (R packages: “mixOmics”, v 6.14.1, and “mdatools”, v0.14.0). Features with Variable Importance in Projection (VIP) scores ≥2 were considered candidate metabolites related to the PD bacterial score. As we observed metabolites and pathways potentially influenced by diet and vitamin intake, we additionally adjusted for a healthy diet, represented by a high score on the 2015 Healthy Eating Index (HEI), and vitamin B intake in sensitivity analyses in the subset of patients with available dietary data (n = 90). In addition, as we observed potentially altered lipid metabolism and given the potential influence of lipid-lowering medications on circulating lipids, we further adjusted for lipid-lowering medication use (yes versus no) in sensitivity analyses among patients with medication use data available (n = 109).

We additionally performed an exploratory analysis using pairwise Spearman correlations to examine associations between the 29 metabolic features identified in the PLS model and 31 PD-related bacterial genera. This univariate approach was intended to provide preliminary insight into specific metabolite–genus relationships. However, given the limited sample size, we acknowledge that this analysis is underpowered; therefore, the results are considered to provide supplementary support to the primary findings only.

### 2.5. Pathway enrichment analysis

To identify perturbed metabolic pathways associated with the PD bacterial score, we conducted pathway enrichment analysis using *mummichog* [16], implemented in MetaboAnalyst 6.0, an online platform for metabolomics data analysis [17]. All 4762 features were included in this pathway enrichment analysis. *mummichog* is a well-validated algorithm for untargeted metabolomics analysis. It bypasses the identification of metabolites and predicts their functional activities (represented by KEGG pathways) by weighing the collective contributions of metabolites within a pathway or network. To identify enriched pathways, within each permutation, *mummichog* calculates Fisher’s exact test statistics by comparing the number of significant hits to the expected number of metabolites hits in each pathway. Gamma p-values are then calculated based on permutation tests. We entered the feature table in mixed mode (both positive and negative columns included) and set the input parameters to a 20 ppm error tolerance for *m/z* and default adduct type (M[1+], *M* + H[1+], *M*+Na[1+]) for 100 permutation tests.

## 3. Results

The demographic and other relevant clinical and biosample characteristics of all 113 study participants with PD are provided in Table 1. The majority of the PD patients were white (75 %) and male (69 %). The mean age at blood sample collection was 70.5 years and 72.4 years for fecal sample collection. The mean duration between fecal and serum sample collection was 2 years with a range from 0 to 6.8 years. The average duration since PD diagnosis was 8 years and on average, they were treated with 550 mg of levodopa per day. Their mean score on Unified Parkinson’s Disease Rating Scale was 29.3, and a majority of patients (89.4 %) had a Hoehn & Yahr staging score less than 3. Among PD patients with available dietary data (n = 90), the mean daily vitamin B intake (from diet and supplements) was 2.1 mg, and the mean HEI score was 63.8.

In total, 4762 features (2716 in HILIC column and 2046 in C18 column) were detected in HRM. Among these, 266 features (153 in HILIC column and 113 in C18 column) were associated with the PD bacterial score with a VIP ≥2. We successfully annotated 83 features and present 29 features with a p-value <0.1 in Table 2. Among these 29 features, 12 belong to the lipids and lipid-like molecules superclass, 5 to organoheterocyclic compounds, 4 to benzenoids, 2 to phenylpropanoids and polyketides, 3 to organic acids and derivatives, 2 to organic oxygen compounds, and 1 to alkaloids and derivatives. From the putative compound list, for each feature, we identified compounds with potential for anti-inflammation (3 features), pro-inflammation (1 feature), being neuroactive (4 features), and of microbial origin (5 features). The full list of 266 features can be found in Supplementary Table S3. The correlation matrix for metabolite and bacterial genus can be found in Supplementary Figure S2.

**Fig. 2.**
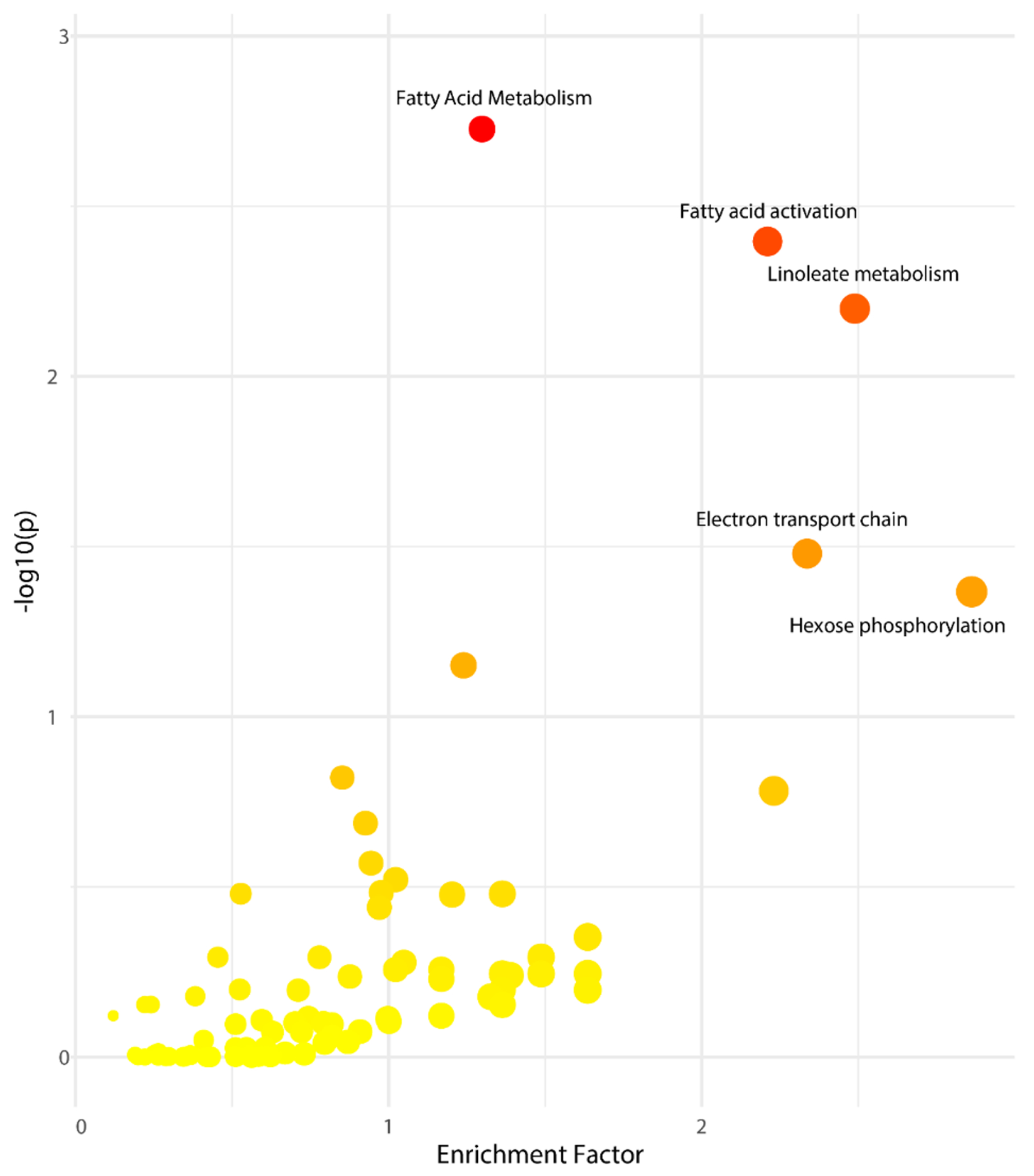
Enriched Metabolic Pathways Associated with PD-related Microbiome. Scatter plot of enriched pathway provided by mummichog. The color and size of each data point correspond to −log10(Fisher P-value) and the enrichment factor (the ratio between the number of significant peaks and the expected number of peaks within that pathway) of each pathway, respectively.

Pathway enrichment analysis using *mummichog* indicated perturbed metabolic function associated with the PD bacterial score to be related to lipids and energy metabolism, such as fatty acid activation and metabolism, linoleate metabolism, carbohydrate metabolism (including hexose phosphorylation, starch and sucrose metabolism), phospholipid metabolism, and vitamin B6 (pyridoxine) metabolism (Table 3, Fig. 2).

Lipids and lipid-like molecules carried greater weight among the metabolomic features identified by our PLS regression. Pathway enrichment analysis (via *mummichog*) further indicated enrichment in pathways related to fatty acid activation and metabolism, de novo fatty acid biosynthesis, linoleate metabolism, and glycerophospholipid metabolism. Together, these results suggest altered lipid metabolism to be most prominently associated with PD-related gut bacterial genera in PD patients.

The serum metabolome of our PD patients suggests that linoleate metabolism enrichment is associated with PD-related gut bacteria, and this is further supported by PLS regression-identified putative linoleic acid (LA, *m/z* = 281.25) being marginally associated with PD bacterial score (coefficient = 0.009, 95 % CI: [−0.001, 0.019], VIP=3.84). These results suggest perturbations in LA metabolism, which holds a multifaceted yet critical role in innate immune response and neuroactivity.

We identified enrichment of glycerophospholipid and glycosphingolipid metabolism pathways in PD patients, and one glycerophospholipid, phosphoethanolamine (PE) (*m/z* = 818.562), associated with the PD bacterial score (β = −0.006, 95 % CI [−0.01, −0.002]). In addition, its intensity was weak-to-moderately correlated with the abundance of three Ruminococcaceae genera: DTU089 (*r* = −0.21, abundance decreased in PD), Ruminiclostridium_5 (*r* = −0.25, abundance increased in PD) and one unspecified Ruminococcaceae genus (*r* = −0.21, abundance increased in PD); one Lachnospiraceae: Lachnospiraceae_UCG-004 (*r*= 0.24, abundance decreased in PD), and one Tannerellaceae: Parabacteroides (*r* = −0.21, abundance increased in PD) (Figure. S2). In addition, we identified a negative association between serum levels of putative monoacylglycerides (MG(20:4/0:0), *m/z* = 377.27) and the PD bacterial score (β = −0.005, 95 % CI [−0.008, −0.003]), as well as a positive association for MG(16:1/0:0) (*m/z* = 329.268) with the PD bacterial score (β = 0.006, 95 % CI [0.001, 0.011]).

We conducted sensitivity analyses adjusting separately for HEI score (Table S4–S5, Figure. S3), vitamin B intake (from food and supplements, Table S6−S7, Figure. S4), and lipid-lowering medication use (Table S8−S9, Figure. S5). In all sensitivity analyses, the results indicating lipid metabolism perturbation and immune response persisted. In the sensitivity analysis adjusting for vitamin B intake the association between serum vitamin B1 (thiamine) and the PD-bacterial score was partially attenuated and enrichment in the vitamin B6 metabolism pathway decreased.

## 4. Discussion

In this study, we examined the relationship between serum metabolome and gut microbiome in a subset of participants from the PEG studies. We integrated our metabolomic and microbiome data to identify metabolites that are associated with a PD bacterial score we generated. These metabolites indicate that the PD-related gut microbiome may be associated with enriched lipid metabolism and potential immune modulation observed in PD patients.

The importance of lipid dysregulation in PD has been widely studied [18], yet there are currently few studies that assess the contributions of gut microbiota to lipid dysregulation, even though gut bacteria are critical for human lipid metabolism. The microbiome produces, digests, and utilizes lipids that reach the gastrointestinal (GI) tract [19]. Conversely, lipids that enter the GI tract can also shape the composition and abundance of gut bacteria. For example, certain free fatty acids can perturb bacterial cell membranes, inhibit bacterial growth, and even trigger bacterial death [20].

Linoleic acid (LA) is an essential polyunsaturated omega-6 fatty acid that can only be obtained from the diet and is further metabolized in the gut. LA and its metabolites play an essential role in humans, as they have anti-inflammatory property and might be neuroprotective in PD. However, excessive LA and its downstream metabolites (e.g., arachidonic acid, prostaglandin) in the linoleate metabolism pathway have also been associated with inflammation. A small case-control study reported that PD patients have lower LA levels in serum and that LA levels are inversely associated with motor severity [21]. Our findings suggest an association between the gut microbiome and altered linoleic acid metabolism in PD patients. A targeted analysis of LA and its metabolites in a larger number of PD patients, along with gut microbiome profiling, may further elucidate this relationship.

Based on serum samples from PEG study participants, we previously found that our patients had a perturbed glycerophospholipid metabolism compared to controls [13]. Our current results suggest that this may be related to gut bacterial abundance in PD. Glycerophospholipids, or phospholipids, are the main components of cell membranes and are critical for maintaining the integrity of the lipid bilayers of cells and organelles, as well as being involved in cell functions such as endocytosis or mitophagy. In addition, they act as signaling molecules and regulate lipid metabolism-related gene expression. Our results suggest that gut bacteria associated with PD are associated with altered serum phospholipid levels in PD patients. Other phospholipids have been associated with PD and gut bacterial composition previously. For example, an observational study of 63 PD patients and 61 healthy controls identified several glycerophospholipids associated with *Roseburia, Lactobacillus*, and *Akkermansia* in PD patients [8]; a hospital-based lipidomic study of 28 PD patients and 18 controls found upregulated plasma levels of LysoPC(18:2) and PA(18:2/15:0) in PD patients [22]; a larger hospital-based study of 170 PD patients and 120 controls reported decreased phosphatidylcholine PC(34:2), PC(46:2), PE(34:2), and PS (40:4) in PD patients [23]. While these results suggest that lipid metabolism is dysregulated in PD patients, it remains to be determined how specific lipids and their associated pathways are affected.Previous studies were small and their results were heterogeneous, possibly due to differences in study design, lab protocols used for biosample analyses, or population-specific differences such as race, age, and stage of PD.

Glycerolipids, which are metabolically closely related to glycerophospholipids, may also play a role in the pathophysiology of PD. Abnormal metabolism of glycerolipids, specifically monoacylglycerol, has been suggested in in-vitro studies and animal models of PD. A change in the expression of monoacylglyceride lipase has been observed in the substantia nigra of PD patients, and it has been suggested that inhibition of MAG lipase, resulting in higher levels of monoacylglycerides, may be neuroprotective in PD [24,25]. With respect to the involvement of the microbiome, *Akkermansia muciniphila* has been linked to increased levels of monoacylglycerols, such as 2-arachidonoylglycerol (2-AG), 2-oleoylglycerol (2-OG), and 2-palmitoyl-glycerol (2-PG), which have anti-inflammatory properties and are beneficial for gut integrity [19]. However, we observed only a weak correlation between monoacylglyceride (*m/z* = 377.27 and *m/z* = 329.268) and *Akkermansia* genus abundance (*r*= 0.1 and *r*= 0.02, respectively) in our study. Much remains unknown about lipid metabolism in PD, especially regarding connections to the gut microbiome, and further studies are warranted.

Our earlier findings indicated that PD patients, in general, have a lower HEI score (reflecting poorer dietary quality) compared to controls, and that specific gut microbiome taxa are differentially abundant due to HEI differences within PD patients [26]. For example, microbial genera associated with PD in this analysis, such as *Eggerthella* and *Tyzzerella_4*, were also associated with HEI among PD patients [26]. In addition, HEI, as an indicator of dietary quality, may also directly influence host metabolism (reflected by the serum metabolome), which provided the rationale for adjusting for dietary quality in our sensitivity analyses. Given the potential influence of diet on both the gut microbiome and host metabolism, we conducted sensitivity analyses adjusting for dietary quality (HEI) to better understand the observed associations. After adjustment, lipid metabolism perturbation persisted, indicating that the connection between PD-associated bacteria and lipid metabolism may be independent of diet. In terms of a PD-specific gut microbiome, perturbed lipid metabolism stands out from all dysregulated metabolic activities in PD patients. As an additional sensitivity analysis, we adjusted for lipid-lowering medication use to assess whether they contributed to the observed alterations in lipid metabolism, but the overall findings remained as reported. Thus, we speculate that the gut microbiome of PD patients is involved in various lipid metabolism activities and is associated with some of the difference in circulating lipids in PD, as suggested by our pathway enrichment analysis and the differences in serum levels for individual lipid molecules we observed related to our PD microbiome score.

We observed enrichment in vitamin B6 (pyridoxine) metabolism and elevations in serum vitamin B1 levels in association with the PD bacterial score (Table 3). Since vitamin B is primarily derived from dietary intake, adjusting for vitamin B intake helps to assess whether the observed associations are partially attributable to diet or persisted beyond dietary influences.

When we adjusted for dietary and supplemental vitamin B intake, the association between serum vitamin B1 (thiamine) and the PD-bacterial score was partially attenuated, and the enrichment in vitamin B6 metabolism pathways decreased. This indicates that vitamin B intake from diet is at least partially accountable for the association between PD-related gut bacteria and host vitamin B metabolism. Furthermore, this may imply upregulated neuroinflammation in PD patients, as vitamins B2 and B6 serve as cofactors in the kynurenine pathway (KP), which has been linked to neuroinflammation due to the production of both neuroprotective and neurotoxic metabolites from tryptophan [27,28]. A deficiency in vitamins B2 or B6 shifts the KP toward reduced production of neuroprotective metabolites, such as kynurenic acid (KA), and increased production of neurotoxic metabolites, including 3-hydroxykynurenine (HK) and quinolinic acid (QA) [29,30]. Moreover, the ratio of these blood metabolites (e.g. HK:KA) has been proposed as a biomarker for vitamin B6 deficiency status [31]. Humans cannot synthesize vitamin B, thus, the primary sources are diet and supplements. Additionally, many gut bacteria can produce B vitamins, while others act as consumers. Therefore, gut bacteria may mediate vitamin B metabolism and inflammation in PD patients, as the gut microbiota composition in PD patients differs from non-PD individuals. We propose that dietary vitamin B intake and an increased abundance of B vitamin-producing bacteria may benefit PD patients by modulating inflammation. Future studies investigating vitamin B biosynthesis and consumption activities in the gut microbiome of PD patients could further elucidate the connection between vitamin B metabolism and PD through the gut microbiota.

Our pilot study is currently one of the largest human studies to investigate metabolome-microbiome associations in PD in an untargeted manner. Several challenges limit the interpretation of our results, though we anticipate that ongoing research will progressively address these gaps. First, our pilot sample of 113 PD patients is relatively small for analyzing high-dimensional data with sufficient statistical power. Another challenge is the still limited annotation of metabolomics data. We set a relatively high error tolerance of *m/z* and rt, which will inevitably compromise the accuracy of the annotation. The microbiome data presents similar challenges, as we had to rely on genus taxonomy level in the analysis due to the limited species information in the reference database. Thus, we cannot assess the distinctive metabolic function of bacterial species. However, whole genome sequencing techniques, such as shotgun sequencing, could effectively address this limitation. In addition, given the cross-sectional and observational nature of this study, the observed microbiome metabolome relationships should be interpreted as associations rather than evidence of causality or biological directionality. Separately, many of the fecal and serum samples from participants were collected at different time points, with a time lapse exceeding one year in ~60 % of our participants. This may have only allowed us to identify patterns and relationships that are relatively persistent. In support of the stability of the fecal microbiome, a study of 37 healthy adults collected fecal samples 2 to 13 times for up to 296 weeks apart and found that individual microbiota were fairly stable, with 60 % of all strains remaining stable over the course of 5 years [32]. Another study that focused on the metabolome collected serum samples from 157 subjects on two occasions and found that more than half of the features in their untargeted metabolomics analysis remained stable after 100 days [33]. Therefore, we likely identified only the most stable associations between the microbiome and the metabolome.

## 5. Conclusion

In summary, by integrating the gut microbiome and serum metabolome in an untargeted manner, we identified some intriguing connections between a disrupted metabolism of lipids, potential immune disturbances, and potentially vitamin B-mediated regulation of kynurenine pathway metabolism in PD, which appear to be related to a PD-specific gut microbiome. Future studies with both targeted and untargeted approaches to identify neuroactive metabolites of microbial origin may be warranted to advance neurodegenerative disease treatments, as the gut-brain axis is increasingly recognized as playing an essential role in PD.

## Supplementary Material

1

Supplementary material associated with this article can be found, in the online version, at doi:10.1016/j.dscb.2026.100310.

## Figures and Tables

**Table 1:** Characteristics of PD patients included in the analysis from the PEG Study (n= 113).

	PD (n = 113)
**Racial minority**	
White	85 (75.2 %)
Non-white	28 (24.8 %)
**Sex**	
Male	78 (69.0 %)
Female	35 (31.0 %)
**Age at stool collection**	
Mean (SD)	72.4 (9.5)
**Age at blood collection**	
Mean (SD)	70.5 (9.5)
**Years between blood and stool collection**	
Mean [Min, Max]	2.0 [0, 6.8]
**Sequencing platform**	
HiSeq	86 (76.1 %)
MiSeq	27 (23.9 %)
**PD duration (year)**	
Mean (SD)	8.0 (4.40)
**Hoehn-Yahr staging at stool collection**	
<3	101 (89.4 %)
≥3	12 (10.6 %)
**UPDRS III score at stool collection**	
Mean (SD)	29.3 (13.3)
**Levodopa daily dosage (mg)**	
Mean (SD)	548 (461)
**Total vitamin B6 intake (mg)**	
Mean (SD)	2.1 (1.1)
Missing	23
**Total HEI score (2015 version, range 0–100)**	
Mean (SD)	63.8 (9.3)
Missing (N)	23
**Lipid-lowering medication use**	
Yes	43 (38.0 %)
No	66 (58.0%)
Missing (N)	4 (4.0%)

PEG: Parkinson’s, Environment and Gene study; UPDRS III: Unified Parkinson’s Disease Rating Scale Park III; HEI: Healthy Eating Index.

**Table 2:** Putatively annotated metabolic features associated with PD-related genera from the partial least square regression^1^.

m/z	Time	Coefficient (95 % CI)	Adjusted p-value	VIP	Column	Putative Compound^2^	Superclass*
169.086	284.667	0.012 (0.002, 0.023)	3.20E-02	5.19	HILIC	135-Trimethoxybenzene; (4-Hydroxy-3-methoxyphenyl)ethanol; 2-Furanylmethyl butanoate; Ethyl 2-furanpropionate; Epoxyoxophorone; 1-Ipomeanol; 4-Ipomeanol; 26-Dimethoxy-4-methylphenol	Benzenoids
464.191	45.647	0.012 (−0.001, 0.026)	7.40E-02	5.14	HILIC	Dihydroisomorphine-6-glucuronide§; Etodolac acyl glucuronide†; Dihydromorphine-3-glucuronide§; Dihydroisomorphine-3-glucuronide§	Alkaloids and derivatives
221.077	124.674	0.011 (0.003, 0.018)	1.20E-02	4.52	HILIC	Brassitin; Serinyl-Aspartate; Aspartyl-Serine¶; Dehydroxyzyleuton; L-beta-aspartyl-L-serine	Organoheterocyclic compounds
180.102	282.421	0.01 (0.001, 0.019)	3.40E-02	4.33	HILIC	Salsolinol§ 3,4-Methylenedioxyamphetamine§; 23-Dihydro-5-(3-hydroxypropanoyl)-1H-pyrrolizine; 123,456-Hexahydro-5-(1-hydroxyethylidene)-7H-cyclopentabpyridin-7-one; Ethyl N-methylanthranilate; 35-Dimethylphenyl methylcarbamate; Maltoxazine; N-methylphenylalanine; alpha-Methylphenylalanine; (*R*)-Salsolinol§; 2(N)-Methyl-norsalsolinol§	Organoheterocyclic compounds
377.196	38.244	−0.01 (−0.021, 0.001)	7.90E-02	4.32	HILIC	16alpha-hydroxyprednisolone; 12alpha-Hydroxy-1318-dehydroparain; 111,214-Trihydroxy-7-methoxy-81,113-abietatrien-206-olide; 18-Oxocortisol	Lipids and lipid-like molecules
281.247	292.307	0.009 (−0.001, 0.019)	7.00E-02	3.84	HILIC	5-Octadecynoic acid; Ethyl 2E4Z-hexadecadienoate; Linalyl caprylate† Mangiferic acid; Linoelaidic acid†; 10E12Z-Octadecadienoic acid; 9E11E-Octadecadienoic acid; Bovinic acid†; Linoleic acid†	Organic oxygen compounds
195.138	281.560	−0.009 (−0.018, 0.001)	8.00E-02	3.63	HILIC	(22-Diethoxyethyl)benzene; (*S*)-Neolyratyl acetate; 1-(2-Furanyl)-1-octanone; 8-Ocimenyl acetate; Menthadienyl acetate; Carvyl acetate; (*R*)-2-Acetoxy-p-mentha-18-diene; Perillyl acetate; (*S*)-p-Mentha-18-dien-10-yl acetate; (2S4R)-p-Mentha-1(7)5-dien-2-ol acetate; Ethyl (2E4Z7Z)-Decatrienoate; Ethyl (2E4E7Z)-Decatrienoate; ()-Myrtenyl acetate; Neocnidilide; Hexylresorcinol; (SZ)-Lyratol acetate; 4-Hydroxypropofol	Phenylpropanoids and polyketides
151.087	38.410	0.008 (0.001, 0.016)	4.20E-02	3.60	HILIC	2-Acetyl-35-dimethylpyrazine; 2-Acetyl-36-dimethylpyrazine; 78-Dihydro-3-methylpyrrolo12-apyrimidin-2(6H)-one; 2-Acetyl-3-ethylpyrazine; 6-Methylnicotinamide¶	Lipids and lipid-like molecules
351.215	38.794	−0.008 (−0.018, 0.002)	9.90E-02	3.41	HILIC	51218R-TriHEPE; Oryzalic acid B; Oryzalic acid A; (ent-6alpha7alpha12alpha)-6712-Trihydroxy-16-kauren-19-oic acid; PGH3; 20-oxo-leukotriene B4‡ 15-Oxo-lipoxin A4† 15-Epi-lipoxin B5†; 12-Oxo-20-hydroxy-leukotriene B4‡ Resolvin E1† 15-Keto-prostaglandin E2† Prostaglandin D3† Prostaglandin E3† 8-iso15-keto-PGE2†	Lipids and lipid-like molecules
285.099	55.495	−0.008 (−0.015, −0.001)	3.80E-02	3.37	HILIC	D-Vacciniin; 2-O-Benzoyl-D-glucose; p-Cresol glucuronide¶	Organoheterocyclic compounds
311.221	35.693	−0.007 (−0.012, −0.002)	9.00E-03	3.15	HILIC	Auxin b; Sterebin A; (9Z11R12S13S15Z)-1213-Epoxy-11-hydroxy-915-octadecadienoic acid; 12(13)Ep-9-KODE	Organic acids and derivatives
177.055	284.086	0.007 (−0.002, 0.016)	9.90E-02	3.11	HILIC	4-Methylumbelliferone; 3-(34-Methylenedioxyphenyl)propenal; 7-Hydroxy-6-methyl-2H-1-benzopyran-2-one; 145-Naphthalenetriol; 10-Hydroxy-28-decadiene-46-diynoic acid; Herniarin	Organoheterocyclic compounds
193.159	277.008	0.006 (0.001, 0.012)	3.90E-02	2.67	HILIC	alpha-Ionone; Phenethyl isoamyl ether; 4-(266-Trimethyl-13-cyclohexadien-1-yl)-2-butanone; Vitispirane; 2457alpha-Tetrahydro-1447a-tetramethyl-1H-inden-2-ol; beta-Ionone; Cycloionone; alpha-Damascone; Isospirene; (*R*)-(E)-47-Megastigmadien-9-one; delta-Damascone; gamma-Ionone; Edulan I; (E)-58-Megastigmadien-4-one; (2E4Z7Z)-247-Tridecatrienal; 4-(266-Trimethylcyclohex-1-enyl)but-2-en-4-one; Pseudoionone; 4-(4-Methyl-3-pentenyl)-3-cyclohexene-1-carboxaldehyde; 23-Diisopropyl-5-methylphenol; 24-Diisopropyl-3-methylphenol; 24-Diisopropyl-5-methylphenol; 25-Diisopropyl-3-methylphenol; 25-Diisopropyl-4-methylphenol; 26-Diisopropyl-3-methylphenol	Organic oxygen compounds
329.268	25.720	0.006 (0.001, 0.011)	2.30E-02	2.56	HILIC	Avocadene 4-acetate; Avocadene 2-acetate; Avocadene 1-acetate; MG(16:1(9Z)0:00:0)§¶; MG(0:016:1(9Z)0:0)§¶	Organoheterocyclic compounds
818.562	66.356	−0.006 (−0.01, −0.002)	4.00E-03	2.55	HILIC	PE(22:6/20:1); PE(22:5/20:2); PE(22:5/20:2); PE(22:4/20:3); PE(22:4/20:3); PE(22:2/20:5); PE(20:5/22:2); PE(20:3/	Lipids and lipid-like
						22:4); PE(20:3/22:4); PE(20:2/22:5); PE(20:2/22:5); PE(20:1/22:6)	molecules
151.148	26.651	−0.006 (−0.013, 0.001)	8.30E-02	2.52	HILIC	(E)-48-Dimethyl-137-nonatriene; Cystophorene	Benzenoids
270.154	33.759	0.006 (−0.001, 0.013)	9.50E-02	2.48	HILIC	2-(4-Methyl-5-thiazolyl)ethyl octanoate	Lipids and lipid-like molecules
377.270	237.921	−0.005 (−0.008, −0.003)	1.00E-03	2.44	C18	Persenone A; 12-Gingerol; 1-Acetoxy-2-hydroxy-51,215-heneicosatrien-4-one; MG(20:4/0:0)§¶; MG(0:0/20:4)§¶; 2-Arachidonylglycerol	Benzenoids
585.486	238.061	−0.005 (−0.009, −0.001)	1.90E-02	2.32	C18	Erythrinasinate A	Lipids and lipid-like molecules
247.144	52.941	−0.005 (−0.011, 0.001)	7.40E-02	2.24	HILIC	Lenticin	Lipids and lipid-like molecules
266.121	44.626	0.005 (0, 0.01)	6.30E-02	2.17	HILIC	Thiamine	Lipids and lipid-like molecules
149.027	101.243	−0.005 (−0.01, 0)	3.90E-02	2.16	HILIC	2-Oxo-4-methylthiobutanoic acid	Organic acids and derivatives
107.047	97.901	−0.005 (−0.01, 0)	6.10E-02	2.14	HILIC	Benzaldehyde	Lipids and lipid-like molecules
343.123	109.031	0.005 (−0.001, 0.011)	9.00E-02	2.09	HILIC	Sucrose; D-Maltose; Melibiose	Benzenoids
405.267	267.942	−0.004 (−0.009, 0)	3.90E-02	2.08	C18	(6b7bl3R)-67-Diacetoxy-814-labdadiene-13-ol; Annocherin A; 3-Oxocholic acid; 37-Dihydroxy-12-oxocholanoic acid; 7-Ketodeoxycholic acid	Phenylpropanoids and polyketides
162.056	40.608	0.004 (−0.001, 0.009)	8.10E-02	2.05	C18	3 - Methyl di oxyi ndole	Lipids and lipid-like molecules
333.280	253.338	−0.004 (−0.008, 0)	4.10E-02	2.03	C18	(all-Z)-71,013-Docosatrienoic acid; Docosatrienoic acid	Organic acids and derivatives
289.194	215.286	−0.004 (−0.009, 0.001)	7.50E-02	2.01	C18	3-hydroxyropivacaine; Verapamil metabolite D-617	Lipids and lipid-like molecules
288.119	128.322	0.005 (0.001, 0.008)	1.90E-02	2.00	HILIC	N-Ribosylhistidine	Lipids and lipid-like molecules

1 Selected feature that were successfully annotated, with VIP>2 and adjusted p-value <0.1.

2 Most features have multiple compounds match based on m/z and time. The full list of potential compounds can be found in Supplementary table S2.

* Classification according to the Human Metabolome Database (HMDB).

† Anti-inflammatory;

‡ pro-inflammatory;

§ Neuroactive:

¶ microbial origin;.

**Table 3:** Top enriched pathways associated with PD-related microbiome from mummichog.

Pathway	Pathway total # of Metabolites	Total # of Hits	Statistically Significant # of Hits	Expected # of Hits	P(Fisher) *	P(EASE) *	P (Gamma) *
Fatty Acid Metabolism	63	21	10	7.7	0.002	0.007	0.024
Fatty acid activation	74	61	20	9.1	0.004	0.009	0.024
Linoleate metabolism	46	39	14	5.6	0.006	0.016	0.024
Hexose phosphorylation	20	19	7	2.5	0.043	0.114	0.028
Starch and Sucrose Metabolism	33	13	5	4.0	0.071	0.198	0.032
De novo fatty acid biosynthesis	106	51	12	12.9	0.205	0.315	0.038
N-Glycan biosynthesis	48	16	5	5.9	0.151	0.329	0.039
Electron transport chain	7	2	2	0.9	0.033	0.331	0.039
Vitamin B6 (pyridoxine) metabolism	11	8	3	1.3	0.165	0.442	0.048
Prostaglandin formation from dihomo gama-linoleic acid	11	8	3	1.3	0.165	0.442	0.048
Glycosphingolipid metabolism	67	36	8	8.2	0.328	0.490	0.052
Phosphatidylinositol phosphate metabolism	59	32	7	7.2	0.363	0.539	0.057
Phytanic acid peroxisomal oxidation	34	21	5	4.2	0.333	0.548	0.058
Di-unsaturated fatty acid beta-oxidation	26	10	3	3.2	0.269	0.567	0.060
Glycosphingolipid biosynthesis - ganglioseries	62	16	4	7.6	0.331	0.578	0.061
Fructose and mannose metabolism	33	31	6	4.0	0.509	0.690	0.078
N-Glycan Degradation	16	6	2	2.0	0.301	0.701	0.080
Drug metabolism - cytochrome P450	53	50	9	6.5	0.576	0.714	0.083
Omega-3 fatty acid metabolism	39	26	5	4.8	0.527	0.723	0.085
Glycerophospholipid metabolism	156	58	10	19.1	0.633	0.754	0.091

* Fisher p-values was calculated based on Fisher’s exact test; EASE p value is a more stringent modified Fisher’s exact test p-value; Gamma p-value was calculated based on permutation tests that randomly resamples the reference list to create a Gamma null distribution.

## Data Availability

The microbiome data used in the study are available on National Center for Biotechnology Information BioProject (Project ID: PRJNA1101026). The metabolomic data used are available on Metabolomics Workbench (Study ID: ST003159). Further data are available upon request from the corresponding author.
